# Comparative analysis of vaginal microbiota sampling using menstrual cups and high vaginal swabs in pregnant women living with HIV-1 infection

**DOI:** 10.3389/fcimb.2023.1190160

**Published:** 2023-05-09

**Authors:** Charlotte-Eve S. Short, Rachael Quinlan, Yun S. Lee, Veronica G. Preda, Ann Smith, Julian R. Marchesi, Robin Shattock, Phillip R. Bennett, David A. MacIntyre, Graham P. Taylor

**Affiliations:** ^1^ Section of Virology, Department of Infectious Disease, Imperial College London, London, United Kingdom; ^2^ St Mary’s Hospital, Imperial College Healthcare NHS Trust, London, United Kingdom; ^3^ March of Dimes Prematurity Research Centre, Division of the Institute of Reproductive and Developmental Biology, Department of Metabolism, Digestion, and Reproduction, Imperial College London, London, United Kingdom; ^4^ Faculty of Health and Applied Sciences, University West of England, Bristol, United Kingdom; ^5^ Marchesi Laboratory, Department of Metabolism, Digestion, and Reproduction, Division of Digestive Disease, Faculty of Medicine, Imperial College London, London, United Kingdom; ^6^ Section of Immunology of Infection, Department of Infectious Disease, Imperial College London, London, United Kingdom

**Keywords:** menstrual cup, swab, cervicovaginal fluid, sampling, microbiota, metataxonomics, HIV

## Abstract

**Background:**

Menstrual cups (MCs) are increasingly used to collect cervicovaginal secretions to characterise vaginal mucosal immunology, in conjunction with high vaginal swabs (HVS) for metataxonomics, particularly in HIV transmission studies. We hypothesised that both methods of collecting bacterial biomass are equivalent for 16S rRNA gene sequencing.

**Material and Methods:**

Cervicovaginal fluid (CVF) samples from 16 pregnant women with HIV-1 (PWWH) were included to represent the major vaginal bacterial community state types (CST I-V). Women underwent sampling during the second trimester by liquid amies HVS followed by a MC (Soft disc™) and samples were stored at -80°C. Bacterial cell pellets obtained from swab elution and MC (500 µL, 1 in 10 dilution) were resuspended in 120 µL PBS for DNA extraction. Bacterial 16S rRNA gene sequencing was performed using V1-V2 primers and were analysed using MOTHUR. Paired total DNA, bacterial load, amplicon read counts, diversity matrices and bacterial taxa were compared by sampling method using MicrobiomeAnalyst, SPSS and R.

**Results:**

The total DNA eluted from one aliquot of diluted CVF from an MC was similar to that of a HVS (993ng and 609ng, p=0.18); the mean bacterial loads were also comparable for both methods (MC: 8.0 log10 16S rRNA gene copies versus HVS: 7.9 log10 16S rRNA gene copies, p=0.27). The mean number of sequence reads generated from MC samples was lower than from HVS (MC: 12730; HVS:14830, p=0.05). The α-diversity metrices were similar for both techniques; MC Species Observed: 41 (range 12-96) versus HVS: 47 (range 16-96), p=0.15; MC Inverse Simpson Index: 1.98 (range 1.0-4.0) versus HVS: 0.48 (range 1.0-4.4), p=0.22). The three most abundant species observed were: *Lactobacillus iners*, *Lactobacillus crispatus* and *Gardnerella vaginalis*. Hierarchical clustering of relative abundance data showed that samples obtained using different techniques in an individual clustered in the same CST group.

**Conclusion:**

These data demonstrate that despite sampling slightly different areas of the lower genital tract, there was no difference in bacterial load or composition between methods. Both are suitable for characterisation of vaginal microbiota in PWWH. The MC offers advantages, including a higher volume of sample available for DNA extraction and complimentary assays.

## Introduction

The importance of microbiota in shaping health and disease states is increasingly appreciated. In the lower reproductive tract, a cervicovaginal microbiota dominated by one or relatively few *Lactobacillus* species is thought to promote reproductive health by inhibiting inflammation and preventing overgrowth of certain pathobionts implicated in several genital tract pathologies including bacterial vaginosis, vaginitis (including aerobic, inflammatory and atrophic) (Donders et al., 2017; Stewart et al., 2022), pelvic inflammatory disease, cervical intraepithelial neoplasia (Norenhag et al., 2020), vulval dermatological conditions (Brunner et al., 2021), menstrual disorders (Chen et al., 2021) and poor pregnancy outcomes such as recurrent miscarriage (Grewal et al., 2022) and preterm birth (Gudnadottir et al., 2022). A loss of *Lactobacillus* species from this niche is also a key risk factor for sexual transmission of infections including *Chlamydia trachomatis (* Ceccarani et al., 2019) and HIV (Bayigga et al., 2019).

There are now many techniques for study of the vaginal microbiota, in addition to standard traditional Gram staining and bacterial culture, both of which still play important clinical roles. Molecular techniques enable identification of organisms that are difficult to culture and include nucleic acid amplification of species-specific genes, often used for diagnostic purposes, and qPCR for total bacterial load or species-specific abundance. Sequencing techniques enable characterisation of bacterial communities from amplified DNA libraries of varying fragment lengths, either targeted to regions of the bacterial 16S rRNA genes (metataxonomics) (Marchesi and Ravel, 2015), multiple loci, or non-targeted whole genome sequencing (Malla et al., 2018). Metataxonomics has a low cost and error rate, but it can only accurately characterise to the genus level whereas multiple gene loci or whole genome sequencing can offer species and strain level and functional gene information including antimicrobial resistance.

Several sampling techniques are available to collect cervicovaginal secretions for onward assays, but there is no consensus on the optimum method for microbiota characterisation. High vaginal swabs (HVS) are widely used to sample secretions from the posterior fornix and vaginal wall (MacIntyre et al., 2015; Short et al., 2020). These swabs can be made from several materials such as cotton, polyester, rayon, nylon and even ophthalmic grade sponge with potential differences in absorption and elution (Castle et al., 2004; Dezzutti et al., 2011). Alternative sampling techniques historically used for sampling the lower female genital tract include saline lavage and absorbent wicks and tampons (Snowhite et al., 2002; van de Wijgert et al., 2006; Dezzutti et al., 2011; Jespers et al., 2011). Other sampling devices used for the characterisation of vaginal microbiota include cervical brushes (Mitra et al., 2017), some of which have been developed for self-sampling (Virtanen et al., 2017). The choice of sampling method may depend on what complementary assays are planned and pre-processing requirements (Jespers et al., 2011). For example, cytobrushes have been compared with HVS and have been demonstrated to be an effective method for sampling the cervical surface for metataxonomic, with the advantage of also providing host cellular material for flow cytometry (Mitra et al., 2017).

Menstrual cups (MCs) can collect large volumes of un-diluted cervicovaginal fluid (CVF), which can also be used for diagnostic vaginal microscopy. They provide the option of self-sampling and are increasingly used for studying the microbiota functional and immune interactions (Masson et al., 2019; Short et al., 2020; Wu et al., 2022) but have not been compared head to head with the more commonly used HVS for metataxonomic and bacterial load estimation. In this paper we compare a MC (Soft disc™) with a polyester HVS for DNA yield and metataxonomics to identify different vaginal microbiota taxa and community structures in a group of pregnant women with HIV-1 infection. We hypothesise that the two methods are equivalent, and both are valid for characterisation of the lower genital tract microbiota.

## Materials and methods

Paired samples from a subgroup of sixteen pregnant women living with HIV from the Immunological Basis of Preterm Delivery Study were used for these analyses, on the basis that all major community state types (CSTs) would be represented, as identified, from previous metataxonomic analysis, from the same samples (Short et al., 2020). The size of sample set was chosen on the basis of both the breath of CSTs, as well as the number of available paired samples and as such no formal sample size calculation was performed. This study was approved by the Southeast Coast RES Committee (13/LO/0107). Written informed consent was obtained enabling clinical data and sample collection. Inclusion criteria were: known HIV-1 antibody status; confirmed singleton pregnancy (by ultrasound); age > 18 and ability to provide informed consent. Exclusion criteria were: multiple or *in-vitro* fertilization pregnancy, injecting drug use and CD4 count < 350 cells/µL. Data on medical, obstetric and drug history were recorded including smoking status, antiretroviral drug exposure and recent antibiotic use. The practice of vaginal douching, recent sexual intercourse and vaginal pH were recorded. Screening for *Syphilis*, *Gonorrhoea* and *Chlamydial* infection was routinely offered as per national guidelines (Gilleece et al., 2019).

### Sample collection

During attendance at routine second trimester antenatal appointments women were invited to donate both MC and HVS samples. The sampling procedure was clinician or self-taken HVS of the high lateral vaginal wall with a Liquid Amies Swab (BBL™ CultureSwab™, BD) followed by clinician or self-insertion of a MC (Soft disc™, The Flex Company, previously manufactured as Instead Soft cup™) for a minimum of five minutes, transferred to a sterile 50mL plastic conical tube. Samples were immediately transferred to the laboratory on wet ice and were stored at -80°C. CVF was removed from the MC prior to further processing by thawing on ice for a maximum of 30 minutes and centrifugation at 4°C for 15 mins at 400 x *g* to separate the CVF from the MC into the base of the conical tube, as previously described (Cosgrove et al., 2016; Short et al., 2018). CVF was divided into 100-200µL aliquots in 1.5mL microtubes, using a positive displacement pipette (Rainin c10–100™, Mettler Toledo) for the handling of high viscosity fluids. For DNA extraction, CVF was diluted 1 in 10 with an extraction buffer consisting of 1X protease cocktail I (Calbiochem™, 539131, Merk), 10µL 10% Sodium Azide solution, 0.75 g NaCl, final volume made to 50mL with 1X phosphate buffer solution (PBS) filter sterilised (Castle et al., 2004; Short et al., 2018). Bacterial biomass from swabs were extracted and pelleted as previously described (MacIntyre et al., 2015).

### DNA extraction

Bacterial cell pellets obtained from both swab and 500 µL of diluted CVF were resuspended in filter sterilised PBS to a volume of 120 µL. DNA extraction was performed using a combination of enzymatic digestion and mechanical disruption of cell membranes and QIAamp Pathogen mini kits (Qiagen), eluted in AVE buffer to a final volume of 100 µL, as previously described (MacIntyre et al., 2015). DNA concentrations were measured using a Qubit™ high sensitivity kit (Thermo Fisher Scientific).

### Quantitative polymerase chain reaction

qPCR was carried out for quantification of 16S rRNA gene copy number to compare the bacterial load collected by each technique. qPCR was performed with universal BactQUANT 16S rRNA gene primers (Forward primer:5′-CTACGGGAGGCAGCA, Reverse primer: 5′-GGACTACCGGGTATCTAATC) (Sigma) with the FAM labelled BactQUANT probe ((6FAM) 5′-CAGCAGCCGCGGTA-3′ (MGBNFQ)) (Liu et al., 2012; Mitra et al., 2017) on a CFX Real Time PCR system (Bio-Rad). A tenfold standard curve (3030 to 303,039,700 copies) of *Escherichia coli* genomic DNA (Sigma, D4889) was generated, each reaction contained 5 µL of DNA sample or standard, 10 µL Platinum PCR Super mix UDG containing Rox (Life Tech, 11730-017) and primers and probe. Thermal cycling was performed at 3 min at 50°C for UNG incubation,10 min at 95°C for Taq activation, then 40 cycles of 15 s at 95°C for denaturation and 1 min at 60°C for annealing and extension. Cycle threshold (Ct) value for each reaction were obtained using CFX Maestro software version 1.1 (Bio-Rad) after application of fluorescence drift correction, background subtraction using the ‘curve fit’ option and automatic Ct baseline definition. Ct values were converted to 16S rRNA gene copy number from the generated standard curve. Samples were run in duplicate; a negative control of molecular grade water was included to eliminate contamination.

### Metataxonomics

DNA concentrations were unadjusted prior to library preparation (range 1ng/µL to 36ng/µL, total volume 20µL). The V1-V2 hypervariable regions of the 16S rRNA gene were amplified with a fusion primer set that includes four different 28F primers chosen to improve detection of *Bifidobacteriales* (including the *Gardnerella* genus) and a 388R primer (Frank et al., 2008). The 28F-YM forward primer (5′-GAGTTTGATCNTGGCTCAG-3′) was mixed in a ratio of 4:1:1:1 with 28F Borrellia (5′-GAGTTTGATCCTGGCTTAG-3′), 28F Chloroflex (5′-GAATTTGATCTTGGTTCAG-3′), and 28F Bifido (5′-GGGTTCGATTCTGGCTCAG-3′) (RTL Genomics Amplicon Diversity Assay List). The forward primers included an Illumina i5 adapter (5′-AATGATACGGCGACCACC GAGATCTACAC-3′), an 8-base-pair (bp) bar code and primer pad (forward, 5′-TATGGTAATT-3′). The 388R reverse primer (5′-TGCTGCCTCCCGTAGGAGT-3′) was constructed with an Illumina i7 adapter (5′-CAAGCAGAAGACGGCATACGAGAT-3′), an 8-bp bar code and a primer pad (reverse, 5′-AGTCAGTCAG- 3′). The pair end multiplex sequencing was performed on an Illumina MiSeq platform (Illumina Inc.) at Research and Testing Laboratory (Lubbock, TX, USA).

The 16S rRNA sequences were analysed using the MiSeq SOP pipeline with the MOTHUR software package (Kozich et al., 2013). Highly similar amplicons were clustered into operational taxonomic units (OTUs) using the kmer searching method and the Silva bacterial database (www.arb-silva.de/) (Quast et al., 2013). All OTUs had a taxonomic cut-off of ≥97%. Classification was performed using the Ribosomal Database Project (RDP) reference sequence files and the Wang method (Wang et al., 2007). The RDP MultiClassifier script was used for determination of OTUs (phylum to genus) and species level taxonomies were determined using USEARCH (Edgar, 2010). OTUs with <10 reads across the dataset were considered rare taxa and were grouped (taxonomy_species X). Diversity indices (Inverse Simpson index and species observed (SObs) were calculated using the Vegan package within R (Dixon, 2003).

### Statistical analyses

The total DNA concentration extracted by sampling method was calculated by multiplying the original sample DNA concentration in ng/µL by the eluted volume of 100 µL. The total bacterial load extracted by sampling method was calculated by multiplying the number of 16S rRNA gene copies in a reaction volume of 5µL by a factor of twenty and is reported as total copies/100µL. The total DNA quantity, bacterial load and unrarefied OTU read sequences, sample richness (SObs) and α diversity (Inverse Simpson Index) were compared by sampling method using the paired t test in SPSS (Version 28.0, IBM). A p-value less than 0.05 was considered statistically significant.

Bacterial taxon data visualisation and statistical analyses were performed in MicrobiomeAnalyst and STAMP packages (Parks and Beiko, 2010; Chong et al., 2020). Beta diversity profiling of sampling method dissimilarity was explored using a Principal Coordinate Analysis plot of rarefied taxon data with Bray-Curtis dissimilarities distances and PERMANOVA. Community State Types (CSTs) were compared in individuals by method on unrarefied Centred Log Ratio transformed data using Ward hierarchical clustering with average distances using the top 25 species observed, accounting for >95% of the total reads. CSTs were compositionally consistent with those originally described by Ravel (Ravel et al., 2011) and Gajer et al. (2012): CST I: *L. crispatus* dominance; CST II: *L. gasseri* dominance; CST III *L. iners* dominance; CST IV-A: Moderate *L. iners* with mixed anaerobes; CST IV-B: Mixed anaerobes including higher proportions of genus *Atopobium* and BV associated bacteria and CST V: *L. jensenni* dominance (Gajer et al., 2012). Taxon specific abundance by method was compared to identify any species or genus with significant over representation with linear discriminant analysis (LDA) effect size (LEfSe) analysis. A logarithmic LDA score cut off of 2 was used to determine any discriminative features.

## Results

The median age of the 16 participants was 34 years (IQR 30-37). Fourteen were of Black race (88%), two were White. Median gestational age at sampling was 24 weeks (IQR-21-28). All, but one woman, who delivered at 36.9 weeks, went on to have term deliveries. Median CD4 count was 550 cells/mcL (IQR 411-631) and median HIV viral load at baseline was < 40 copies reflecting the fact that 11/16 women conceived on antiretroviral therapy (ART). Two of the 5 women who initiated ART during pregnancy had started prior to the second trimester sampling timepoint. Most women received Non-Nucleoside Reverse Transcriptase Inhibitor based ART, 4 received Integrase Strand Transfer Inhibitor based ART, 2 received Protease Inhibitor based ART and 2 initiated triple Nucleoside Reverse Transcriptase Inhibitor based ART.

### Swabs and menstrual cup yield similar DNA concentrations and bacterial load

Total DNA extracted by each collection method was comparable with similar mean DNA concentration extracted from the cell pellet from one aliquot of diluted MC CVF compared to a HVS (993ng and 609ng, p=0.18), see **Table 1**. The mean total bacterial load was similar for both methods (MC: 8.0 log10 16S rRNA gene copies/100µL versus HVS: 7.9 log10 16S rRNA gene copies/100µL, p=0.27). The number of sequence reads from swab samples were higher than diluted MC samples (median HVS sequence reads: 14830 (range 9572-21793) vs. MC: 12730 (range 7738-18295), p=0.05).

**Table 1:** Comparison of DNA concentration, 16S rRNA gene copies, sequence reads and metataxonomic profiles by collection method.

Method	High vaginal swab	Menstrual cup	P value
**Total DNA concentration eluted/ng** **(mean (range))**	**609 (10-1740)**	**993 (10-3550)**	**0.18**
**Total Bacterial load by method/log10 16S rRNA gene copies/100µL (mean(range))**	**7.9 (6.3-8.3)**	**8.0 (6.8-8.9)**	**0.27**
**Total high-quality sequence reads (mean (range))**	**14830 (9572-21793)**	**12730 (7738-18295)**	**0.05**
**a diversity matrices** **(mean (range))** **Species observed** **Inverse Simpson Index**	**47 (16-96)** **1.9 (1.0-4.4)**	**41 (12-96)** **1.8 (1.0-4.0)**	**0.15** **0.22**
**Community state type (n(%))** **I** **II** **III** **IV-A** **IV-B** **V**	**3 (19)** **1 (6)** **4 (25)** **3 (19)** **3 (19)** **2 (12)**	**3 (19)** **1 (6)** **4 (25)** **3 (19)** **3 (19)** **2 (12)**	**1.0**

### Swabs and menstrual cups provide comparable 16S rRNA gene sequencing results

**Figure 1:**
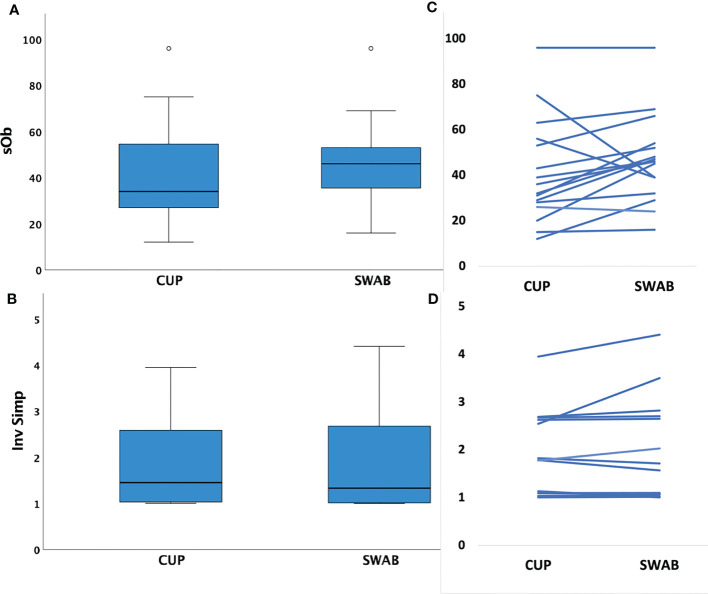
Box plots of mean species richness and alpha diversity indices by method and paired values for individuals. **(A)** Mean species observed (richness, SOb) did not differ by the two different sampling methods (p=0.15, paired t test); **(B)** paired samples showing similar SOb by method in individuals **(C)** Mean Inverse Simpson index (alpha diversity metric) did not differ by collection method (p=0.22, paired t test); **(D)** paired samples showing similar Inverse Simpson indices by method in individuals.

Sample richness was similar in the number of species observed (SOb) by sampling method (mean HVS: 47 (range 16-96) versus MC: 41 (range 12-96), p=0.15). Inverse Simpson Index scores were also similar for samples obtained through each technique (mean HVS: 1.9 (range 1.0-4.4) versus MC: 1.8 (IQR 1.0-4.0), p= 0.22), see **Figure 1**.

**Figure 2:**
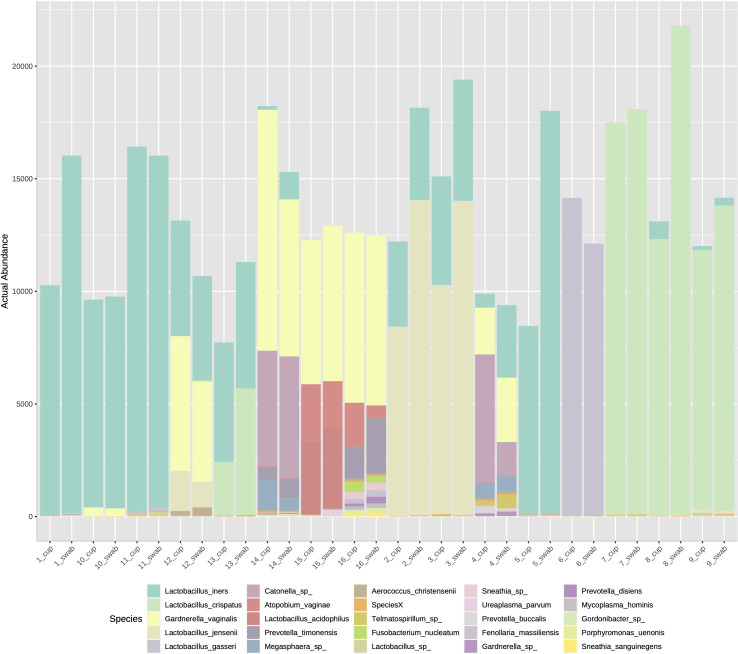
Stacked bar chart of species abundance by sample collection method in individuals 1-16. The top abundant species were *L. iners, L. crispatus* and *G. vaginalis* with no difference on species composition by sampling type.

**Figure 3:**
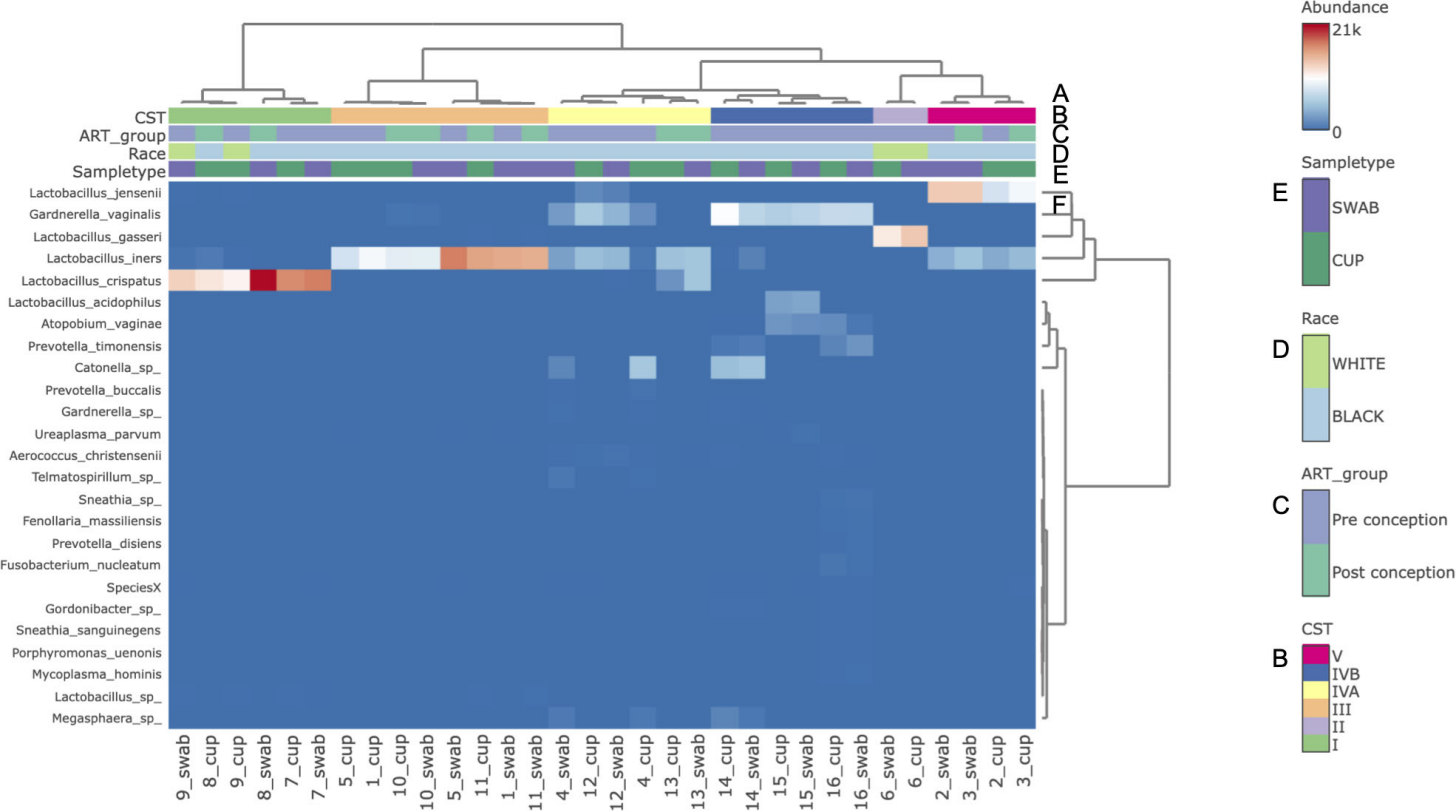
Bacterial species composition of vaginal CSTs from individual pregnant mothers with HIV during the second trimester by sampling method. **(A)** Hierarchical clustering analysis using ward linkage and maximum distances of microbial species data show both methods identified that there were 6 major vaginal microbiota groups; **(B)** CST types: I- *Lactobacillus crispatus* dominant, II- *L. gasseri* dominant, III- *L. iners* dominant, IV-A- moderate *L. iners* with mixed anaerobes and IV-B- mixed anaerobes including higher proportions of genus Atopobium and bacterial vaginosis associated bacteria; **(C)** ART exposure in relation to conception; **(D)** Race; **(E)** Sample type; **(F)** Heat map of relative species abundance of vaginal bacteria with top 25 abundant species shown.

**Figure 4:**
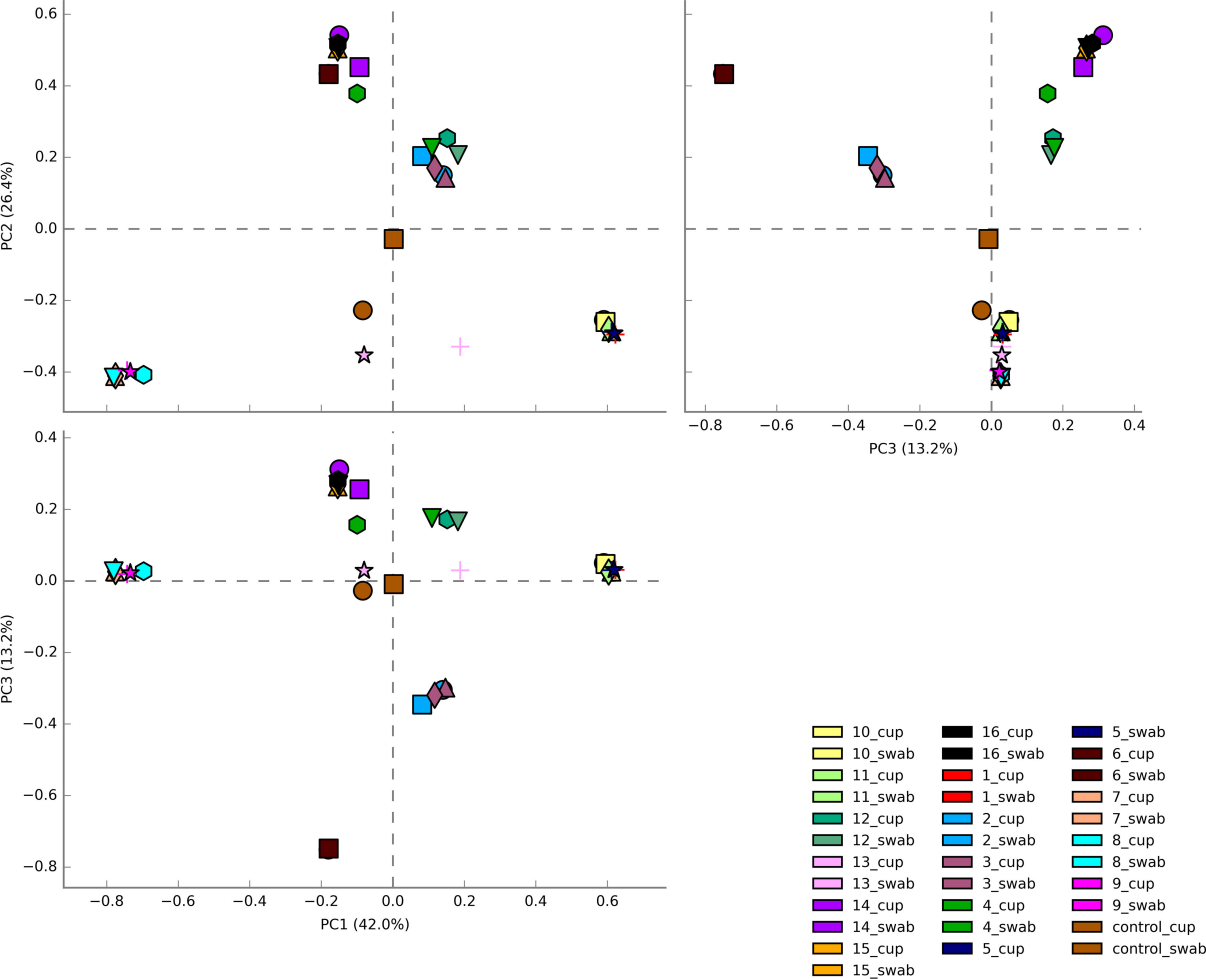
Principal Coordinate analysis (PCoA) plots of beta diversity of all vaginal microbiota samples from HIV-1 infected pregnant women. Samples are coloured by individual patient and sample type are represented by different shapes. Vaginal community diversity and composition largely clustered according to the individual patient with component 1 explaining 42% of variation and component 2 explaining 26% and component 3 explaining 13%.

The three most abundance species were in order: *L. iners*, *L. crispatus* and *G. vaginalis*, see **Figure 2**. Hierarchical clustering of relative abundance data showed that bacterial profiles obtained from the same individual via different samples methods clustered together in the same CST group, see **Table 1** and **Figure 3**. Visualisation of the dissimilarity matrix with PCoA plots for the different sampling methods revealed near identical vaginal community structures with diversity and composition clustering according to patient, PERMANOVA F-value: 0.11861; R-squared: 0.0039381; p-value: 0.998, see **Figure 4**. No differentially abundant taxa were identified in either sampling method by LEfSe analysis, data not shown.

## Discussion

We have demonstrated that MC sampling of the lower female genital tract secretions for DNA extraction and 16S rRNA gene amplicon sequencing provides comparable results to polyester HVS sampling. Whilst these analyses are drawn from a small sample set of pregnant women with HIV, these data are reassuring that MC and other similar methods for collecting undiluted cervicovaginal secretions should perform as well as HVS for metataxonomic and potentially metagenomic techniques and should be generalisable to vaginal sampling from all women.

The trend towards a slightly higher total DNA concentration with the MC method is likely to be the result of a greater starting volume of undiluted CVF collected with the Soft disc™ compared to swab samples which usually require elution. The 1 in 10 dilution of CVF was selected from our group’s previous work with immunoassays as it enabled ease of sample handling, concentrations of related cytokine to be accurately measured as well as producing similar sized cell pellets to the swab method (Short et al., 2018; Short et al., 2020). In a previous study of HIV negative pregnant women we found that MC samples, *in situ* for a minimum of 5 minutes and a maximum of 1 hour, yield a median weight of 0.5g of undiluted CVF which is equivalent to 500µL (Short et al., 2018), similar to others experience in non-pregnant women (Boskey et al., 2003; Jaumdally et al., 2017).

Undiluted CVF is very viscous and can require a positive displacement pipette for direct processing. Some groups have previously been deterred by this handling issue (Chappell et al., 2014), however, with the correct pipette, or with direct addition of elution buffer into the conical tube containing the MC (Jaumdally et al., 2018), the final volume can be significant. For example, 500µL of CVF diluted 1 in 10 would give a 5mL starting volume from which DNA could be extracted. This could potentially yield ten-fold the quantity of DNA albeit with the limitations of the yield of the chosen DNA purification procedure (Mattei et al., 2019). This dilution is easily adjusted, DNA concentration standardised prior to library preparation, with significant remaining available material for additional assays.

The Soft disc™, which opens inside the vagina to its 7cm diameter (Boskey et al., 2003), also collects CVF from a larger surface area of vaginal epithelium than a HVS. In spite of the potential for the MC samples to be more representative of the total bacterial biomass of lower genital tract, the lack of difference found between the methods may indicate the similarity in microbiota between the surface of cervix, high and middle vagina, replicating the finding of others (Chen et al., 2017).

In our experience, the MC was acceptable and well-tolerated by study participants and can be used to self-sample the lower female genital tract, potentially away from a healthcare facility which could offer some logistical benefits. Self-sampled vaginal swabs have been previously shown to be comparable to physician taken swabs for vaginal microbiota sequencing studies, which makes such methods ideal for large scale field studies (Forney et al., 2010). However, time taken to store sample should always be minimised and standardised to reduce any impact on sample integrity. Quick storage into freezers is particularly important for metabolomic work, yet there is data from faecal microbiota samples that shows that with storage at ambient temperatures, interpatient variability is maintained at forty-eight hours, including in the *Lactobacillaceae* and *Bifidobacteriaceae* bacterial families commonly found in the vagina (Nel Van Zyl et al., 2020).

Our understanding of the importance of vagina microbiota to health and disease is expanding rapidly with the use of culture independent DNA assays. Key to this is our understanding of how bacteria interact with their human host through allied techniques such as: flow cytometry of local immune cell populations (Byrne et al., 2021); multiplex immune assays of cytokines and other immune proteins (Short et al., 2020; Short et al., 2021); RNA sequencing of the bacterial transcriptome (Mohd Zaki et al., 2022); mass-spectrometry of bacterial metabolomic signatures and their glycan binding proteins (Pruski et al., 2021; Wu et al., 2022) in addition to a variety of microarrays (Li and Feizi, 2018).

To date, MC sampling of CVF has been widely applied in the study of HIV infection to assess genital HIV viral load measurement (Jaumdally et al., 2017), HIV-1 diffusion (Shukair et al., 2013), microbicide drug concentrations (Price et al., 2011) and to quantify mucosal immunoglobins (Archary et al., 2015; Cosgrove et al., 2016) and cytokines (Archary et al., 2015; Jaumdally et al., 2018). Its utility as a collection method is increasingly recognised in the study of microbiota host interactions (Masson et al., 2019; Short et al., 2020; Wu et al., 2022) but it can now be considered valid for metataxonomic characterisation. This method’s utility may increase as the importance of studying how vaginal microbiota increase HIV transmission risk and risk of preterm birth in women living with HIV are gaining wider recognition and have the potential to be modified through modulating these microbial interactions.

## Conclusion

Ultimately the choice of CVF collection method in trial design will depend on the assays required, their processing requirements, as well as cost, but if MCs are used then additional HVS sampling is not required for DNA based characterisation of the microbiota.

## Data availability statement

The original contributions presented in the study are publicly available. This data can be found here: https://www.ebi.ac.uk/ena/browser/view/PRJEB60624, accession number PRJEB60624.

## Ethics statement

The studies involving human participants were reviewed and approved by Southeast Coast RES Committee (13/LO/0107). The patients/participants provided their written informed consent to participate in this study.

## Author contributions

C-ES and GT conceived and designed the study. Patient recruitment and sample collection were undertaken by C-ES and RQ. Experiments and data collection were performed by C-ES, RQ and VP. Data processing, analyses, and interpretation were performed by C-ES, AS, GT and DM. All figures and tables were generated by C-ES. C-ES wrote the first draft of the manuscript and all authors contributed critical revisions to the paper, interpretation of the results and approved the final version.

## References

[B1] Archary D. Liebenberg L. J. Werner L. Tulsi S. Majola N. Naicker N.. (2015). Randomized cross-sectional study to compare HIV-1 specific antibody and cytokine concentrations in female genital secretions obtained by menstrual cup and cervicovaginal lavage. PloS One 10 (7), e0131906. doi: 10.1371/journal.pone.0131906 26147923 PMC4492781

[B2] Bayigga L. Kateete D. P. Anderson D. J. Sekikubo M. Nakanjako D. (2019). Diversity of vaginal microbiota in sub-Saharan Africa and its effects on HIV transmission and prevention. Am. J. obstetrics gynecol. 220 (2), 155–166. doi: 10.1016/j.ajog.2018.10.014 PMC10715630 30321529

[B3] Boskey E. R. Moench T. R. Hees P. S. Cone R. A. (2003). A self-sampling method to obtain large volumes of undiluted cervicovaginal secretions. Sexually transmitted diseases 30 (2), 107–109. doi: 10.1097/00007435-200302000-00002 12567165

[B4] Brunner A. Medvecz M. Makra N. Sardy M. Komka K. Gugolya M.. (2021). Human beta defensin levels and vaginal microbiome composition in post-menopausal women diagnosed with lichen sclerosus. Sci. Rep. 11 (1), 15999. doi: 10.1038/s41598-021-94880-4 34362937 PMC8346569

[B5] Byrne E. H. Farcasanu M. Bloom S. M. Xulu N. Xu J. Hykes B. L. Jr.. (2021). Antigen presenting cells link the female genital tract microbiome to mucosal inflammation, with hormonal contraception as an additional modulator of inflammatory signatures. Front. Cell Infect. Microbiol. 11, 733619. doi: 10.3389/fcimb.2021.733619 34604114 PMC8482842

[B6] Castle P. E. Rodriguez A. C. Bowman F. P. Herrero R. Schiffman M. Bratti M. C.. (2004). Comparison of ophthalmic sponges for measurements of immune markers from cervical secretions. Clin. Diagn. Lab. Immunol. 11 (2), 399–405. doi: 10.1128/CDLI.11.2.399-405.2004 15013994 PMC371211

[B7] Ceccarani C. Foschi C. Parolin C. D’Antuono A. Gaspari V. Consolandi C.. (2019). Diversity of vaginal microbiome and metabolome during genital infections. Sci. Rep. 9 (1), 14095. doi: 10.1038/s41598-019-50410-x 31575935 PMC6773718

[B8] Chappell C. A. Rohan L. C. Moncla B. J. Wang L. Meyn L. A. Bunge K.. (2014). The effects of reproductive hormones on the physical properties of cervicovaginal fluid. Am. J. obstetrics gynecol. 211 (3), 226 e1–226 e7. doi: 10.1016/j.ajog.2014.03.041 PMC4149850 24662718

[B9] Chen C. X. Carpenter J. S. Gao X. Toh E. Dong Q. Nelson D. E.. (2021). Associations between dysmenorrhea symptom-based phenotypes and vaginal microbiome: a pilot study. Nurs. Res. 70 (4), 248–255. doi: 10.1097/NNR.0000000000000510 33813547 PMC8222084

[B10] Chen C. Song X. Wei W. Zhong H. Dai J. Lan Z.. (2017). The microbiota continuum along the female reproductive tract and its relation to uterine-related diseases. Nat. Commun. 8 (1), 875. doi: 10.1038/s41467-017-00901-0 29042534 PMC5645390

[B11] Chong J. Liu P. Zhou G. Xia J. (2020). Using MicrobiomeAnalyst for comprehensive statistical, functional, and meta-analysis of microbiome data. Nat. Protoc. 15 (3), 799–821. doi: 10.1038/s41596-019-0264-1 31942082

[B12] Cosgrove C. A. Lacey C. J. Cope A. V. Bartolf A. Morris G. Yan C.. (2016). Comparative immunogenicity of HIV-1 gp140 vaccine delivered by parenteral, and mucosal routes in female volunteers; MUCOVAC2, a randomized two centre study. PloS One 11 (5), e0152038. doi: 10.1371/journal.pone.0152038 27159166 PMC4861263

[B13] Dezzutti C. S. Hendrix C. W. Marrazzo J. M. Pan Z. Wang L. Louissaint N.. (2011). Performance of swabs, lavage, and diluents to quantify biomarkers of female genital tract soluble mucosal mediators. PloS One 6 (8), e23136. doi: 10.1371/journal.pone.0023136 21858008 PMC3155537

[B14] Dixon P. (2003). VEGAN, a package of r functions for community ecology. J. Veg Sci. 14 (6), 927–930. doi: 10.1111/j.1654-1103.2003.tb02228.x

[B15] Donders G. G. G. Bellen G. Grinceviciene S. Ruban K. Vieira-Baptista P. (2017). Aerobic vaginitis: no longer a stranger. Res. Microbiol. 168 (9-10), 845–858. doi: 10.1016/j.resmic.2017.04.004 28502874

[B16] Edgar R. C. (2010). Search and clustering orders of magnitude faster than BLAST. Bioinformatics 26 (19), 2460–2461. doi: 10.1093/bioinformatics/btq461 20709691

[B17] Forney L. J. Gajer P. Williams C. J. Schneider G. M. Koenig S. S. McCulle S. L.. (2010). Comparison of self-collected and physician-collected vaginal swabs for microbiome analysis. J. Clin. Microbiol. 48 (5), 1741–1748. doi: 10.1128/JCM.01710-09 20200290 PMC2863907

[B18] Frank J. A. Reich C. I. Sharma S. Weisbaum J. S. Wilson B. A. Olsen G. J. (2008). Critical evaluation of two primers commonly used for amplification of bacterial 16S rRNA genes. Appl. Environ. Microbiol. 74 (8), 2461–2470. doi: 10.1128/AEM.02272-07 18296538 PMC2293150

[B19] Gajer P. Brotman R. M. Bai G. Sakamoto J. Schutte U. M. Zhong X.. (2012). Temporal dynamics of the human vaginal microbiota. Sci. Trans. Med. 4 (132), 132ra52. doi: 10.1126/scitranslmed.3003605 PMC3722878 22553250

[B20] Gilleece D. Y. Tariq D. S. Bamford D. A. Bhagani D. S. Byrne D. L. Clarke D. E.. (2019). British HIV Association guidelines for the management of HIV in pregnancy and postpartum 2018. HIV Med. 20 Suppl 3, s2–s85. doi: 10.1111/hiv.12720 30869192

[B21] Grewal K. Lee Y. S. Smith A. Brosens J. J. Bourne T. Al-Memar M.. (2022). Chromosomally normal miscarriage is associated with vaginal dysbiosis and local inflammation. BMC Med. 20 (1), 38. doi: 10.1186/s12916-021-02227-7 35090453 PMC8796436

[B22] Gudnadottir U. Debelius J. W. Du J. Hugerth L. W. Danielsson H. Schuppe-Koistinen I.. (2022). The vaginal microbiome and the risk of preterm birth: a systematic review and network meta-analysis. Sci. Rep. 12 (1), 7926. doi: 10.1038/s41598-022-12007-9 35562576 PMC9106729

[B23] Jaumdally S. Z. Jones H. E. Hoover D. R. Gamieldien H. Kriek J. M. Langwenya N.. (2017). Comparison of sampling methods to measure HIV RNA viral load in female genital tract secretions. Am. J. Reprod. Immunol. 77 (3), 1–12. doi: 10.1111/aji.12619 PMC6457130 28111861

[B24] Jaumdally S. Z. Masson L. Jones H. E. Dabee S. Hoover D. R. Gamieldien H.. (2018). Lower genital tract cytokine profiles in south African women living with HIV: influence of mucosal sampling. Sci. Rep. 8 (1), 12203. doi: 10.1038/s41598-018-30663-8 30111808 PMC6093917

[B25] Jespers V. Francis S. C. van de Wijgert J. Crucitti T. (2011). Methodological issues in sampling the local immune system of the female genital tract in the context of HIV prevention trials. Am. J. Reprod. Immunol. 65 (3), 368–376. doi: 10.1111/j.1600-0897.2010.00938.x 21199064

[B26] Kozich J. J. Westcott S. L. Baxter N. T. Highlander S. K. Schloss P. D. (2013). Development of a dual-index sequencing strategy and curation pipeline for analyzing amplicon sequence data on the MiSeq illumina sequencing platform. Appl. Environ. Microbiol. 79 (17), 5112–5120. doi: 10.1128/AEM.01043-13 23793624 PMC3753973

[B27] Li Z. Feizi T. (2018). The neoglycolipid (NGL) technology-based microarrays and future prospects. FEBS Lett. 592 (23), 3976–3991. doi: 10.1002/1873-3468.13217 30074246

[B28] Liu C. M. Aziz M. Kachur S. Hsueh P. R. Huang Y. T. Keim P.. (2012). BactQuant: an enhanced broad-coverage bacterial quantitative real-time PCR assay. BMC Microbiol. 12, 56. doi: 10.1186/1471-2180-12-56 22510143 PMC3464140

[B29] MacIntyre D. A. Chandiramani M. Lee Y. S. Kindinger L. Smith A. Angelopoulos N.. (2015). The vaginal microbiome during pregnancy and the postpartum period in a European population. Sci. Rep. 5, 8988. doi: 10.1038/srep08988 25758319 PMC4355684

[B30] Malla M. A. Dubey A. Kumar A. Yadav S. Hashem A. Abd Allah E. F. (2018). Exploring the human microbiome: the potential future role of next-generation sequencing in disease diagnosis and treatment. Front. Immunol. 9, 2868. doi: 10.3389/fimmu.2018.02868 30666248 PMC6330296

[B31] Marchesi J. R. Ravel J. (2015). The vocabulary of microbiome research: a proposal. Microbiome 3, 31. doi: 10.1186/s40168-015-0094-5 26229597 PMC4520061

[B32] Masson L. Barnabas S. Deese J. Lennard K. Dabee S. Gamieldien H.. (2019). Inflammatory cytokine biomarkers of asymptomatic sexually transmitted infections and vaginal dysbiosis: a multicentre validation study. Sexually transmitted infections 95 (1), 5–12. doi: 10.1136/sextrans-2017-053506 30018088

[B33] Mattei V. Murugesan S. Al Hashmi M. Mathew R. James N. Singh P.. (2019). Evaluation of methods for the extraction of microbial DNA from vaginal swabs used for microbiome studies. Front. Cell Infect. Microbiol. 9, 197. doi: 10.3389/fcimb.2019.00197 31245304 PMC6563847

[B34] Mitra A. MacIntyre D. A. Mahajan V. Lee Y. S. Smith A. Marchesi J. R.. (2017). Comparison of vaginal microbiota sampling techniques: cytobrush versus swab. Sci. Rep. 7 (1), 9802. doi: 10.1038/s41598-017-09844-4 28852043 PMC5575119

[B35] Mohd Zaki A. Hadingham A. Flaviani F. Haque Y. Mi J. D. Finucane D.. (2022). Neutrophils dominate the cervical immune cell population in pregnancy and their transcriptome correlates with the microbial vaginal environment. Front. Microbiol. 13, 904451. doi: 10.3389/fmicb.2022.904451 35774454 PMC9237529

[B36] Nel Van Zyl K. Whitelaw A. C. Newton-Foot M. (2020). The effect of storage conditions on microbial communities in stool. PloS One 15 (1), e0227486. doi: 10.1371/journal.pone.0227486 31935223 PMC6959592

[B37] Norenhag J. Du J. Olovsson M. Verstraelen H. Engstrand L. Brusselaers N. (2020). The vaginal microbiota, human papillomavirus and cervical dysplasia: a systematic review and network meta-analysis. BJOG: an Int. J. obstetrics gynaecol. 127 (2), 171–180. doi: 10.1111/1471-0528.15854 31237400

[B38] Parks D. H. Beiko R. G. (2010). Identifying biologically relevant differences between metagenomic communities. Bioinformatics 26 (6), 715–721. doi: 10.1093/bioinformatics/btq041 20130030

[B39] Price C. F. Tyssen D. Sonza S. Davie A. Evans S. Lewis G. R.. (2011). SPL7013 gel (VivaGel(R)) retains potent HIV-1 and HSV-2 inhibitory activity following vaginal administration in humans. PloS One 6 (9), e24095. doi: 10.1371/journal.pone.0024095 21935377 PMC3174146

[B40] Pruski P. Correia G. D. S. Lewis H. V. Capuccini K. Inglese P. Chan D.. (2021). Direct on-swab metabolic profiling of vaginal microbiome host interactions during pregnancy and preterm birth. Nat. Commun. 12 (1), 5967. doi: 10.1038/s41467-021-26215-w 34645809 PMC8514602

[B41] Quast C. Pruesse E. Yilmaz P. Gerken J. Schweer T. Yarza P.. (2013). The SILVA ribosomal RNA gene database project: improved data processing and web-based tools. Nucleic Acids Res. 41 (Database issue), D590–D596. doi: 10.1093/nar/gks1219 23193283 PMC3531112

[B42] Ravel J. Gajer P. Abdo Z. Schneider G. M. Koenig S. S. McCulle S. L.. (2011). Vaginal microbiome of reproductive-age women. Proc. Natl. Acad. Sci. United States America 108 Suppl 1 (Suppl 1), 4680–4687. doi: 10.1073/pnas.1002611107 PMC3063603 20534435

[B43] Short C. S. Brown R. G. Quinlan R. Lee Y. S. Smith A. Marchesi J. R.. (2020). Lactobacillus-depleted vaginal microbiota in pregnant women living with HIV-1 infection are associated with increased local inflammation and preterm birth. Front. Cell Infect. Microbiol. 10, 596917. doi: 10.3389/fcimb.2020.596917 33643930 PMC7905210

[B44] Short C. S. Quinlan R. Bennett P. Shattock R. J. Taylor G. P. (2018). Optimising the collection of female genital tract fluid for cytokine analysis in pregnant women. J. Immunol. Methods 458, 15–20. doi: 10.1016/j.jim.2018.03.014 29625077 PMC5981004

[B45] Short C. S. Quinlan R. A. Wang X. Preda V. G. Smith A. Marchesi J. R.. (2021). Vaginal microbiota, genital inflammation and extracellular matrix remodelling collagenase: MMP-9 in pregnant women with HIV, a potential preterm birth mechanism warranting further exploration. Front. Cell Infect. Microbiol. 11, 750103. doi: 10.3389/fcimb.2021.750103 34912728 PMC8667959

[B46] Shukair S. A. Allen S. A. Cianci G. C. Stieh D. J. Anderson M. R. Baig S. M.. (2013). Human cervicovaginal mucus contains an activity that hinders HIV-1 movement. Mucosal Immunol. 6 (2), 427–434. doi: 10.1038/mi.2012.87 22990624 PMC3732193

[B47] Snowhite I. V. Jones W. E. Dumestre J. Dunlap K. Braly P. S. Hagensee M. E. (2002). Comparative analysis of methods for collection and measurement of cytokines and immunoglobulins in cervical and vaginal secretions of HIV and HPV infected women. J. Immunol. Methods 263 (1-2), 85–95. doi: 10.1016/S0022-1759(02)00038-8 12009206

[B48] Stewart L. L. Vodstrcil L. A. Coombe J. Bradshaw C. S. Hocking J. S. (2022). Prevalence of bacterial vaginosis in postmenopausal women: a systematic review and meta-analysis. Sex Health 19 (1), 17–26. doi: 10.1071/SH21083 35192453

[B49] van de Wijgert J. Altini L. Jones H. de Kock A. Young T. Williamson A. L.. (2006). Two methods of self-sampling compared to clinician sampling to detect reproductive tract infections in gugulethu, south Africa. Sexually transmitted diseases 33 (8), 516–523. doi: 10.1097/01.olq.0000204671.62529.1f 16572041

[B50] Virtanen S. Kalliala I. Nieminen P. Salonen A. (2017). Comparative analysis of vaginal microbiota sampling using 16S rRNA gene analysis. PloS One 12 (7), e0181477. doi: 10.1371/journal.pone.0181477 28723942 PMC5517051

[B51] Wang Q. Garrity G. M. Tiedje J. M. Cole J. R. (2007). Naive Bayesian classifier for rapid assignment of rRNA sequences into the new bacterial taxonomy. Appl. Environ. Microbiol. 73 (16), 5261–5267. doi: 10.1128/AEM.00062-07 17586664 PMC1950982

[B52] Wu G. Grassi P. MacIntyre D. A. Molina B. G. Sykes L. Kundu S.. (2022). N-glycosylation of cervicovaginal fluid reflects microbial community, immune activity, and pregnancy status. Sci. Rep. 12 (1), 16948. doi: 10.1038/s41598-022-20608-7 36216861 PMC9551102

